# Long-Term Neoplastic Risk Associated With Colorectal Strictures in Crohn’s Disease: A Multicenter Study

**DOI:** 10.1016/j.gastha.2024.05.003

**Published:** 2024-05-16

**Authors:** Thomas Hunaut, Laurent Peyrin-Biroulet, Antoine Le Bozec, Adeline Germain, Corinne Gower-Rousseau, Charles Sabbagh, Guillaume Cadiot, Mathurin Fumery

**Affiliations:** 1 Department of Gastroenterology, Reims University Hospital, Université de Champagne-Ardenne, Reims, France; 2 Department of Gastroenterology, Nancy University Hospital, Vandœuvre-lès-Nancy, France; 3 INFINY Institute, Nancy University Hospital, Vandœuvre-lès-Nancy, France; 4 FHU-CURE, Nancy University Hospital, Vandœuvre-lès-Nancy, France; 5 Groupe Hospitalier privé Ambroise Paré - Hartmann, Paris IBD Center, Neuilly sur Seine, France; 6 Division of Gastroenterology and Hepatology, McGill University Health Centre, Montreal, Quebec, Canada; 7 Department of Pharmacy, Reims University Hospital, Université de Champagne-Ardenne, Reims, France; 8 Department of Digestive Surgery, Nancy University Hospital, University of Lorraine, Vandoeuvre-lès-Nancy, France; 9 Research and Public Health Unit, Reims University Hospital, Université de Champagne-Ardenne, Reims, France; 10 Department of Digestive Surgery, CHU d'Amiens Picardie, Amiens Cedex 01, France; 11 UR UPJV 7518, SSPC (Simplification of Surgical Patients Care), Université de Picardie Jules Verne, Amiens, France; 12 Department of Gastroenterology, Amiens University Hospital, and PeriTox, Université de Picardie, Amiens, France

**Keywords:** Crohn’s Disease, Colonic Stricture, Cancer

## Abstract

**Background and Aims:**

While the occurrence of colonic stricture in Crohn’s disease (CD) always raises concerns about the risk of cancer, the neoplastic risk associated with its stricture remains poorly known.

**Methods:**

All consecutive patients with colorectal stricture complicating CD in 3 academic centers between 1993 and 2022 were included in a retrospective cohort. We collected clinical, endoscopic, surgical, and pathology data and information on outcomes. Factors associated with neoplastic stricture were investigated by logistic regression.

**Results:**

A total of 88 patients (median age, 25 [interquartile range {IQR}, 19–37] years and median disease duration 12 [4–19] years) with 96 colorectal strictures were included. Strictures were nonpassable by the scope in 61.4% (n = 54) of cases, 70.5% (n = 62) were ulcerated, and 62.5% (n = 55) were symptomatic. Colonic resection and endoscopic balloon dilatation were needed in 47.7% (n = 42) and 28.6% (n = 12) of patients, respectively. After a median follow-up of 21.5 months (IQR [5.5–46.5]), 7 (8%) patients were diagnosed with neoplasia at the colonic stricture site (colonic adenocarcinoma, n = 5; neuroendocrine carcinoma, n = 1; and B-cell lymphoproliferative neoplasia, n = 1), with a median stricture duration at colorectal neoplasia diagnosis of 0 month (IQR [0.0–5.5]). While neoplastic strictures were diagnosed in older patients (58 vs 39 years), with longer disease duration (18 vs 11 years) and frequent obstructive symptoms (57.1% vs 11.1%), no patient-related or stricture-related factor was associated with neoplastic stricture in multivariate analysis.

**Conclusion:**

Eight percent of patients with colonic stricture complicating CD developed colorectal cancer. Colorectal cancer and stricture were often diagnosed at the same time and we did not report malignant stricture after 1 year of follow-up.

## Introduction

Crohn’s disease (CD) is a chronic and progressive inflammatory disorder of the gastrointestinal tract that can lead to altered quality of life, disability, and irreversible bowel damage.^1^^,^^2^ CD is also associated with an increased risk of colorectal cancer (CRC). The incidence of CRC in CD has been evaluated between 75 and 82 per 100,000 person-years, with an increase of 40%–60% compared to the general population.^3^, ^4^, ^5^ Risk factors for CRC in inflammatory bowel disease include primary sclerosis cholangitis, family history of colonic cancer, personal history of colonic dysplasia, extensive colitis, pseudopolyps, disease duration over 8 years, and colorectal stricture.^6^, ^7^, ^8^, ^9^, ^10^, ^11^, ^12^, ^13^, ^14^ According to international recommendations, screening colonoscopy is recommended after 8 years of evolution, with timing intervals based on the presence of these factors.^15^

The prevalence of colonic stricture in CD has been evaluated between 8% and 13.5%.^10^, ^11^, ^12^, ^13^, ^14^^,^^16^ The occurrence of colonic stricture in CD always raises concerns about the risk for dysplasia/cancer. European Crohn's and Colitis Organisation (ECCO) guidelines recommended a colonoscopy at the diagnosis of colonic stricture with multiple biopsies followed by annual screening colonoscopy,^13^ but there is no consensus for the management of these strictures. Few studies with conflicting results have evaluated the frequency of CRC associated with colonic stricture in CD, and the natural history of colonic stricture in CD is poorly known. While the oldest study reported a CRC risk of 6.8%,^10^ the most recent found a risk of 0.32%.^12^ Moreover, negative endoscopic biopsies are insufficient to rule out neoplastic complication.^16^ Most of these studies are issued from single center, and were also characterized by a low number of included patients, and little information on clinical, endoscopic, and pathological findings.

Therefore, multicenter studies are needed to better understand the risk of colonic stricture-associated neoplasia and to identify CRC risk factors enabling personalized therapeutic strategies at the era of biologics and modern endoscopy.

## Materials and Methods

### Population

This retrospective cohort study was performed in 3 French referral centers (Amiens, Nancy, and Reims University Hospitals) having access to a clinical database including all consecutive adult patients with colonic stricture and CD between 1993 and 2022. Inclusion criteria were (1) age ≥18 years, (2) CD patient, and (3) colonic stricture. We included all patients with new stricture diagnosis which were then subsequently followed with surveillance. The follow-up began at the date of stricture diagnosis. Colonic stricture was defined as a narrowing of the colonic lumen in coloscopy. Anastomotic strictures, terminal ileum strictures, ileocecal valve strictures, and obvious polypoid lesions producing narrowing of the lumen were excluded.

### Data Collection

Clinical, surgical, and pathological data were extracted from patient’s hospital medical records retrospectively, using a standardized questionnaire specifically developed for this study. The following clinical data were gender, age, age at CD diagnosis, history of colonic surgery, localization and phenotype according to Montreal classification,^17^ perianal disease, history of extra-intestinal autoimmune disease, history of primary sclerosing cholangitis, active smoking, personal history of colonic dysplasia, extensive colitis, familial history of CD or colonic cancer, and disease activity at time of surgery based on physicians’ judgment. The following information on colonic stricture was collected: age at diagnosis, disease duration at stricture diagnosis, localization, length (assessed by colonoscopy or cross-sectional imaging), symptomatic character (abdominal pain, constipation, subocclusive or occlusive syndrome), passable with the endoscope, and presence of ulceration. Follow-up was performed until date of death, stricture disappearance, or censored at December 31, 2022. Stricture-related symptoms and therapeutic and endoscopic outcomes were also collected. Presence of dysplasia (low-grade or high-grade dysplasia) or cancer (histological description) was also recorded whenever available.

### Statistical Analysis

Quantitative variables were described as the mean ± standard deviation or median (interquartile range and minimum and maximum) according to the distribution of the variable, and qualitative variables were described as numbers and percentages. Results were analyzed using Student’s *t*-test (mean ± standard deviation comparison) and Fisher’s exact test (percentage comparison), respectively, to describe difference between both groups. Univariate logistic regression model was used to estimate the association between variables and CRC diagnosis. Multivariate logistic regression was achieved with stepwise backward procedure and adjusted on the variables included in the univariate analysis. All analyses were 2-tailed, and variables with *P* values <.05 were considered statistically significant. The study was performed in accordance with the Declaration of Helsinki, Good Clinical Practice, and applicable regulatory requirements. The study was approved by local Ethics Committee (MR00429082023, “CHU de Reims”). All authors had access to the study data and reviewed and approved the final manuscript.

## Results

### Study Population

We identified 88 patients with a total of 96 colorectal strictures between 1993 and 2022. The main characteristics of the population are presented in Table 1. Fifty-three patients (60.2%) were female, the median age and disease duration at stricture diagnosis were 25 (IQR, 19–37) and 12 (IQR, 4–19) years, respectively. CD location at the diagnosis of colonic stricture according to the Montreal Classification was pure colonic disease (L2) in 42% (n = 37) and ileocolonic disease (L3) in 58% (n = 51) of cases. Respectively, 4.5% (n = 4) and 1.1% (n = 1) of patients had a personal or familial history of colonic dysplasia/CRC. One quarter (n = 21, 23.9%) of patients had a penetrating behavior associated to strictures and another quarter (n = 22, 25%) experienced a prior intestinal resection. Previous exposition to 5-aminosalicylates, azathioprine, anti-tumor necrosis factor, and other biologics were observed in 38.6%, 55.7%, 60.3%, and 12.5% of patients, respectively. Only 2 patients had history of primary sclerosis cholangitis (2.3%).

**Table 1:** Characteristics of the Population at the Time of Colonic Stricture Diagnosis

Characteristics	Overall population N = 88	Nonmalignant colorectal stricture N = 81	Malignant colorectal stricture N = 7	*P* value
Female, n (%)	53 (60.2)	49 (60.5)	4 (57.1)	.99
Age at CD diagnosis (median, IQR)	25.0 [19, 37]	25.0 [19, 37]	32.0 [27, 42]	.39
Familial history of IBD (n, %)	8 (9.1)	8 (9.9)	0	.85
Familial history of colorectal cancer, n (%)	1 (1.1)	1 (1.2)	0	.99
Personal history of colonic dysplasia, n (%)	4 (4.5)	4 (4.9)	0	.99
Personal history of intestinal resection for CD, n (%)	22 (25.0)	19 (24.5)	3 (42.9)	.36
Disease topography				.99
L2, n (%)	37 (42.0)	34 (42.0)	3 (42.9)	
L3, n (%)	51 (58.0)	47 (58.0)	4 (57.1)	
Disease phenotype				.87
B2, n (%)	67 (76.1)	61 (75.3)	6 (85.7)	
B3, n (%)	21 (23.9)	20 (24.7)	1 (14.3)	
Perianal CD, n (%)	29 (33.0)	27 (33.3)	2 (28.6)	.99
Extra-intestinal manifestations, n (%)	7 (8.0)	6 (7.4)	1 (14.3)	.45
Active smoking, n (%)	16 (18.2)	16 (19.8)	0	.34
Extensive colitis, n (%)	66 (75.0)	60 (74.1)	6 (85.7)	.67
Primary sclerosis cholangitis, n (%)	2 (2.3)	1 (1.2)	1 (14.3)	.15
Prior medication exposure				
5-ASA, n (%)	34 (38.6)	33 (40.7)	1 (14.3)	.24
Thiopurines, n (%)	49 (55.7)	46 (56.8)	3 (42.9)	.70
Anti-TNFα, n (%)	53 (60.3)	49 (60.5)	4 (57.1)	.99
Vedolizumab, n (%)	5 (5.7)	5 (6.2)	0	.99
Ustekinumab, n (%)	6 (6.8)	4 (4.9)	2 (18.6)	.07
Other, n (%)	13 (14.8)	12 (14.8)	1 (14.3)	.99
Age at stricture diagnosis (median, IQR)	41.00 [31, 54.75]	39.00 [30.5, 53]	58.00 [48.5, 59]	.03
Disease duration before stricture diagnosis; y (median, IQR)	12.0 [4.0; 19.75]	11 [4.0; 19]	18.0 [11.5; 24.0]	.27
Nonpassable stricture, n (%)	54 (61.4)	48 (59.3)	6 (85.7)	.09
Localization				.01
Rectum, n (%)	8 (9.1)	5 (6.2)	3 (42.9)	
Sigmoid, n (%)	25 (28.4)	25 (30.9)	0	
Left colon, n (%)	29 (32.9)	26 (32.1)	3 (42.9)	
Transverse colon, n (%)	13 (14.8)	13 (16.0)	0	
Right colon, n (%)	11 (12.5)	10 (12.3)	1 (14.2)	
Occlusive and/or subocclusive syndrome, n (%)	13 (14.8)	9 (11.1)	4 (57.1)	<10^−2^
Multiple strictures, n (%)	7 (8.0)	7 (8.6)	0	.99
Colonoscopic aspect				.05
Nonulcerated, n (%)	15 (17.0)	15 (18.5)	0	
Ulcerated, n (%)	62 (70.5)	58 (71.6)	4 (57.1)	
Budding, n (%)	5 (5.7)	3 (3.7)	2 (28.7)	
Surgery				.60
Partial colectomy, n (%)	19 (45.2)	17 (47.2)	2 (33.3)	
Total colectomy, n (%)	12 (28.6)	10 (27.8)	2 (33.3)	
Coloproctectomy, n (%)	3 (7.1)	2 (5.6)	1 (16.7)	
Other, n (%)	8 (19.1)	7 (19.4)	1 (16.7)	
Surgery indication				.30
Occlusive syndrome, n (%)	13 (31.0)	10 (27.7)	3 (42.9)	
Dysplasia or neoplasia, n (%)	2 (4.8)	1 (2.8)	1 (16.7)	
Failure of medical treatment, n (%)	12 (28.6)	11 (30.6)	1 (16.7)	
Other, n (%)	14 (33.2)	13 (36.1)	1 (16.7)	

CD, Crohn’s disease; IBD, inflammatory bowel disease; IQR, interquartile range.

### Stricture Characteristics and Their Management

Strictures characteristics are detailed in Table 1. Strictures were not passable by the scope in 61.4% (n = 54) of cases and 70.5% (n = 62) were ulcerated. Multiple strictures were observed in 8% (n = 7) of patients. Strictures had a median length of 5.5 cm (IQR, 3.6–10.0) estimated during colonoscopy or when nonpassable with computed tomography scan or magnetic resonance imaging, and 62.5% (n = 55) of patients had symptomatic strictures. Location of strictures was right colon in 12.5%, transverse colon in 14.8%, left colon in 32.8%, sigmoid in 28.4%, and rectum in 9.1% of patients. Details concerning stricture management are presented in Table 1. Stricture-related surgery was needed in 47.7% (n = 42) and the most frequent type of surgery was partial colectomy (n = 19, 45.2%). Endoscopic balloon dilation was performed in 13.6% (n = 12) of patients.

### Dysplasia and Cancer Associated With Colonic Stricture

After a median follow-up of 21.5 months (IQR, 5.5–46.5), 7 (8%) among 88 patients with colonic stricture were diagnosed with neoplasia located at the colonic stricture site either at time of stricture diagnosis or during follow-up (Figure). Main characteristics of these patients are presented in Table 2. The median stricture duration at colorectal neoplasia diagnosis was 0.0 month (IQR, 0.0–5.5). Four patients were diagnosed with dysplasia or cancer at stricture diagnosis and the others in the following year. In the 7 patients with malignant stricture, 5 had colonic adenocarcinoma (5.7%) (including 1 mucinous adenocarcinoma), 1 had neuroendocrine carcinoma, and 1 B-cell lymphoproliferative neoplasia. At cancer diagnosis, 5 patients had active colonic inflammation documented with biopsies during the colonoscopy. Three patients died during follow-up (adenocarcinomas, n = 2 and neuroendocrine carcinoma, n = 1), 2 patients were still receiving antineoplastic treatment (adenocarcinomas, n = 2), and patient with B-cells lymphoproliferation was considered in remission. Data were missing for the last patient.

**Figure:**
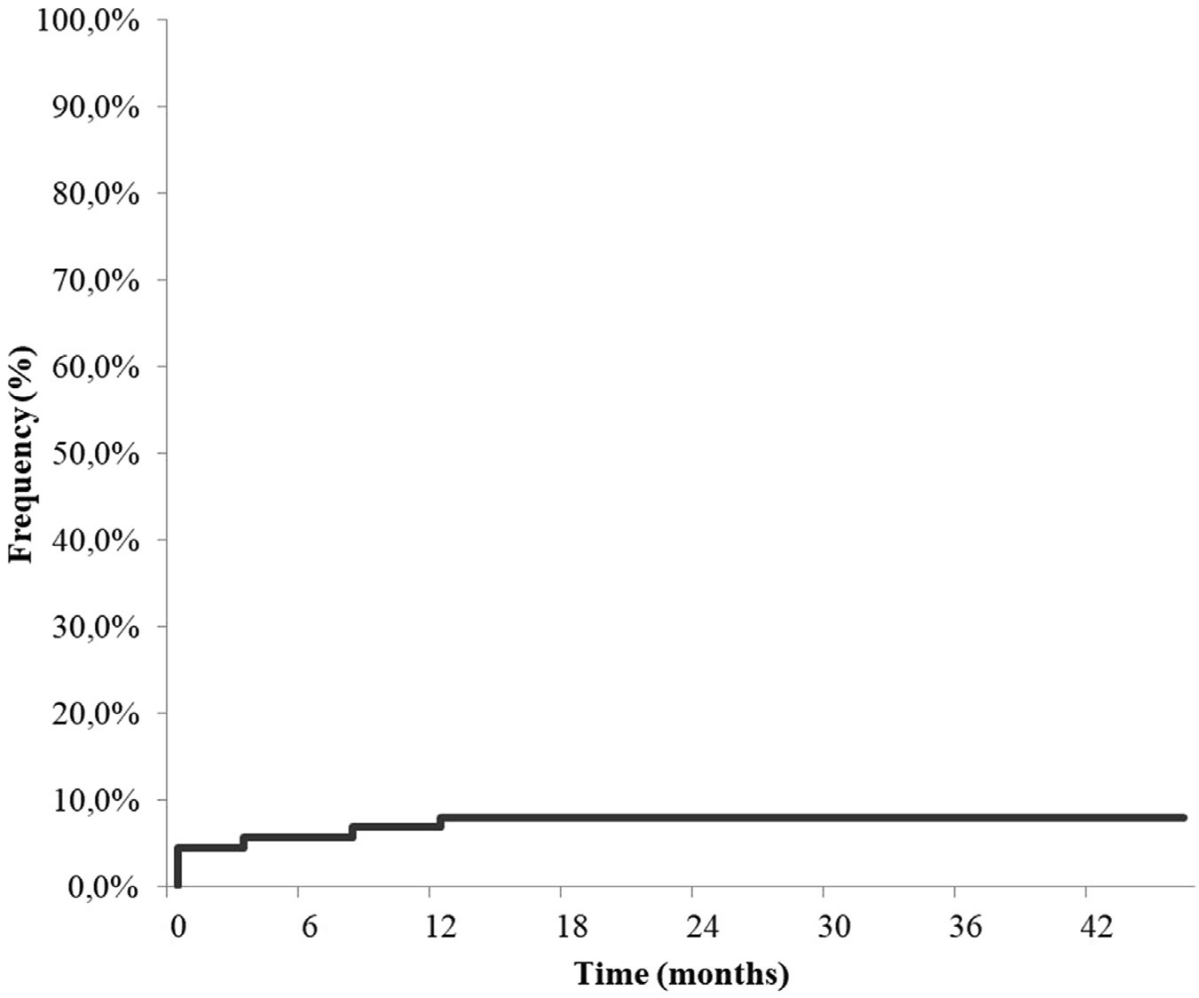
Cumulative risk of malignant stricture in patients with Crohn’s disease.

**Table 2:** Characteristics of Patients With Malignant Stricture

Pathological finding	Moderately to poorly differentiated adenocarcinoma	Mucinous lieberkühnian adenocarcinoma	Dissociated cells adenocarcinoma	Poorly differentiated adenocarcinoma	B-cells lympho-proliferation	Moderately differentiated adenocarcinoma	Neuroendocrine carcinoma
Median stricture duration at cancer diagnosis (month)	3	0	0	12	8	0	0
Localization	Left colon	Rectum	Left colon	Rectum	Rectum	Left colon	Right colon
Occlusive symptoms (yes or no)	Yes	No	Yes	No	No	Yes	Yes
Passable stricture in colonoscopy (yes or no)	No	No	No	No	No	No	No
Disease duration at stricture diagnosis (year)	11	27	18	36	21	12	1
Age at stricture diagnosis (year)	53	58	60	59	32	44	59
Gender	F	F	F	M	M	F	M
Patient	**1**	**2**	**3**	**4**	**5**	**6**	**7**

F, female; M, male.

### Factors Associated With Neoplasia

Neoplastic strictures were diagnosed in older patients than those with benign strictures (median age 58, IQR [48–59] vs median age 39, IQR [30–53] years; *P* = .03) and with disease evolving for longer (median duration 18 years, IQR [11–24] vs median duration 11 years, IQR [4–19]; *P* = .27). Obstructive symptoms were observed in, respectively, 57.1% and 11.1% of patients with neoplastic and benign stricture (*P* < .01). In univariate analysis, obstructive symptoms at stricture diagnosis were associated with neoplastic stricture (odds ratio 10.20, confidence interval 95% [1.96–53.24], *P* < .01). None associated factor with neoplastic stricture was found in multivariate analysis (Table 3).

**Table 3:** Factors Associated With Malignant Colorectal Stricture During in Univariate and Multivariate Analysis

Variable	Univariate analysis OR 95% CI	*P* value	Multivariate analysis OR 95% CI	*P* value
History of primary sclerosis cholangitis	13.33 [0.74–240.70]	.08		
Age at stricture diagnosis	1.07 [0.99–1.15]	.06	1.06 [1.00–1.13]	.06
Stricture duration	0.99 [0.96–1.01]	.30		
Disease duration before stricture diagnosis	1.04 [0.97–1.12]	.25		
Occlusive and/or sub-occlusive syndrome	10.22 [1.96–53.24]	<10^−2^		
Extensive colitis	2.10 [0.24–18.47]	.50		
Ulcerated stricture	0.62 [0.10–3.67]	.60		
Stricture location				
Left colon	Ref			
Rectum	5.20 [0.81–33.56]	.08		
Right colon	0.87 [0.080–9.34]	.91		

CI, confidence interval; OR, odds ratio.

## Discussion

The management of inflammatory bowel disease patients with colonic strictures remains a challenge in clinical practice. The neoplastic risk associated with stricture remains poorly known in the era of biologics and modern endoscopy. In our study, including 88 consecutive patients, the prevalence of malignant stricture was 8%. All these neoplastic lesions were diagnosed at stricture diagnosis or in the following year.

This result is higher than reported in recent studies showing a risk of malignant stricture from 0.32% to 3.5%.^11^, ^12^, ^13^, ^14^^,^^16^ The different types of studied populations could explain these differences. Tilmant et al^18^ included only patients who underwent endoscopic balloon dilatation for colonic stricture. Fumery et al^16^ included only patients operated for a colonic stricture with preoperative negative endoscopic biopsies. Sonnenberg et al^12^ only included patients who underwent colonoscopy with endoscopic biopsies from an administrative database. On the other hand, in a referral study including 175 strictures diagnosed between 1959 and 1985, the prevalence of malignant stricture was 6.8%.^11^

Factors known as associated with malignant colonic strictures are heterogeneous as age, disease duration, short stricture, and absence of active lesion at the time of surgery.^11^^,^^13^ Nevertheless, we did not confirm these findings in our study; disease duration was not significantly associated with cancer risk in multivariate analysis, stricture length could not be analyzed and for most of patients, stricture was described as ulcerated during the colonoscopy. In our study, none factor was associated to malignant stricture in multivariate analysis. Other known risk factors for CRC such as extensive colitis, duration of the disease, and history of primary sclerosis cholangitis were not found as risk factors of cancer.

Among the 7 patients with CRC, diagnosis was made at the time of stricture diagnosis in half of the patients and within the first year for the others. We believe that this result is important for the management of colonic strictures complicating CD in clinical practice. Colonic strictures are considered, since the results of old studies, as a risk factor for CRC, which justifies annual screening endoscopic according to international recommendations. Several hypotheses can be considered: (1) strictures observed are already a neoplastic complication of the colonic inflammatory disease, (2) the stricture itself is a risk factor for cancer on the same site, or (3) the colonic stricture precludes adequate endoscopic surveillance of the whole colon. The fact that all cancers were diagnosed at the time of stricture diagnosis or just after and that none was observed during a median follow-up of 21.5 months argues for the first hypothesis. In Sonnenberg et al^12^ study, duration of follow-up was not mentioned. In Fumery et al^16^ study concerning patient with negative biopsies who underwent colonic stricture surgery, median stricture duration was 6.3 months.

We know that per endoscopic biopsies could not be enough to exclude stricture-associated dysplasia or cancer, explaining potential early use of surgery in these patients.^16^ This risk is however relatively low—2.2%. On the other hand, the absence of longer-term risk of cancer associated with colonic stricture observed in our study is in favor of conservative management in several patients with colonic strictures. In a recent administrative database cohort study, the presence of a colorectal stricture was not independently associated with CRC suggesting for the authors that colonic stricture is a stigma of long-standing active disease rather than an independent risk factor for CRC.^19^

Given the recent evidence on the risk of cancer associated with colonic strictures in CD, systematic colectomy is probably no longer justified. Factors such as a long disease duration, primary sclerosing cholangitis, a history of dysplasia, and nonpassable and/or symptomatic stricture despite endoscopic dilation tend to argue in favor of surgery—especially if limited resection is possible. Conversely, a conservative approach can be considered after a multidisciplinary case-by-case discussion in patients with low risk of neoplastic complications after a precise endoscopic evaluation with systematic biopsies. According to recent recommendations, when the stricture is nonpassable, extending less than 5 cm, endoscopic balloon dilation should be performed after cross-sectional imaging, to pass and explore the upstream colon and screen for dysplasia.^13^ If the stricture becomes passable and ulcerations are observed, intensification of medical therapy could be proposed. According to international recommendations and waiting for news studies, close endoscopic surveillance should be done.

Our study has some limitations. We have to recognize missing data inherent to retrospective studies, especially concerning biological and radiological characteristics. Our sample size was not sufficient to identify factors associated with cancer. Nevertheless, our study has numerous strengths. It is one of the largest studies and duration of follow-up was almost 2 years. Importantly, we included consecutive CD patients followed in 3 French referral centers with colonic stricture regardless of their surgery or endoscopic status.

In conclusion, 8% of patients with colonic stricture complicating CD developed CRC. Cancer and stricture were often diagnosed at the same time and we did not report malignant stricture after 1 year of follow-up. These results plead for a conservative management of passable colonic stricture in CD patients after a precise endoscopic evaluation with biopsies in patients at low risk of CRC.
